# Artificial Intelligence Diagnosis of Parkinson's Disease From MRI Scans

**DOI:** 10.7759/cureus.58841

**Published:** 2024-04-23

**Authors:** Shreya Reddy, Dinesh Giri, Rakesh Patel

**Affiliations:** 1 Biomedical Sciences, Creighton University, Omaha, USA; 2 Research, California Northstate University College of Medicine, Elk Grove, USA; 3 Internal Medicine, East Tennessee State University Quillen College of Medicine, Johnson City, USA

**Keywords:** diagnosis, neurology, mri imaging, artificial intelligence (ai), parkinson’s disease (pd)

## Abstract

Parkinson's disease (PD) is a prevalent neurodegenerative disorder characterized by motor symptoms such as tremors, rigidity, and bradykinesia, affecting approximately 6.1 million people worldwide, according to estimates from the Parkinson's Foundation. Early and accurate diagnosis of PD is crucial for effective management and treatment. In this study, we aimed to develop an artificial intelligence (AI) model capable of distinguishing between magnetic resonance imaging (MRI) scans of individuals with PD and those without PD. A total of 442 MRI scans were utilized for training the AI model, comprising 221 scans of individuals diagnosed with PD and 221 scans of healthy controls. The dataset, obtained from a publicly available image dataset on Kaggle.com, was randomly split into three sets: training, validation, and testing, with 80%, 10%, and 10% of the data allocated to each set, respectively. Leveraging Google's Collaboration platform for model training, the AI model achieved exceptional performance, with accuracy, precision, recall (sensitivity), specificity, and F1-score all measuring at high levels. Additionally, the area under the receiver operating characteristic curve (AUC) for the model was found to be 1, indicating strong discrimination between PD and non-PD cases. This study presents a novel AI model capable of accurately identifying PD from MRI scans with high precision and reliability, offering promise for enhancing early diagnosis and personalized treatment strategies for individuals affected by PD.

## Introduction

Parkinson's disease (PD) stands as one of the most prevalent neurodegenerative disorders worldwide, affecting millions of individuals, particularly those over the age of 60 [1]. It is estimated that approximately 6.1 million people worldwide are living with PD [1]. Characterized by a spectrum of motor symptoms, including tremors, bradykinesia, rigidity, and postural instability, PD not only impairs motor function but also poses significant challenges to cognitive and emotional well-being [2]. The burden of PD extends beyond the individual affected, impacting families, caregivers, and healthcare systems, leading to substantial economic and social costs. Despite extensive research efforts, the precise etiology of PD remains elusive, with a complex interplay of genetic, environmental, and neurobiological factors implicated in its pathogenesis [3]. Understanding the underlying mechanisms driving PD progression is crucial for the development of effective therapeutic strategies aimed at slowing or halting disease progression. Furthermore, the accurate and timely diagnosis of PD presents a considerable clinical challenge, often relying on the assessment of clinical symptoms and neurological examination, which may lack sensitivity and specificity, particularly in the early stages of the disease [4]. Hence, there is an urgent need for reliable biomarkers and diagnostic tools to facilitate early detection and intervention, thereby improving patient outcomes and quality of life.

MRI scans provide valuable insights into structural and functional brain differences between individuals with PD and those without [5]. MRI scans of brains affected by PD often exhibit notable differences compared to those of normal brains, particularly in the frontal lobe and temporal lobe regions [6]. In PD patients, MRI images frequently show cortical thinning and structural alterations in the frontal lobe, which is involved in executive functions, decision-making, and motor planning [7]. Additionally, there may be reduced gray matter volume and altered connectivity patterns in the temporal lobe, which plays a key role in memory formation, language processing, and emotion regulation [8,9]. These changes in the frontal and temporal lobes reflect underlying neurodegenerative processes, including neuronal loss, gliosis, and synaptic dysfunction, contributing to the characteristic motor and non-motor symptoms observed in PD [10]. Conversely, in normal brain MRI scans, the frontal and temporal lobes typically exhibit normal cortical thickness, white matter integrity, and structural organization, without evidence of neurodegenerative changes associated with PD [11]. These differences in MRI characteristics between PD and normal brains underscore the importance of neuroimaging in elucidating the pathophysiology of PD and guiding diagnostic and therapeutic interventions.

In neurology, artificial intelligence (AI) has rapidly emerged as a transformative tool with diverse applications across various domains, revolutionizing the landscape of disease diagnosis, prognosis, and treatment [12]. One prominent area of AI application lies in neuroimaging analysis, where advanced machine learning algorithms are employed to interpret complex imaging data, such as magnetic resonance imaging (MRI), computed tomography (CT), and positron emission tomography (PET) scans [13]. These AI-driven approaches enable the detection of subtle structural and functional abnormalities indicative of neurological conditions, including Alzheimer's disease, PD, multiple sclerosis, and brain tumors, with high accuracy and efficiency [14]. Additionally, AI algorithms have been deployed in the development of predictive models for disease progression and treatment response, leveraging vast datasets to identify patterns and biomarkers associated with disease trajectory [14,15]. Beyond imaging, AI technologies are increasingly utilized in the realm of precision medicine, facilitating the customization of treatment plans based on individual patient characteristics, genetic profiles, and biomarker signatures [16]. Furthermore, AI-driven telemedicine platforms have expanded access to specialized neurological care, allowing for remote consultations, monitoring, and management of patients with neurological disorders, particularly in underserved or remote regions [17]. As AI continues to advance, its integration into neurology holds immense promise for improving diagnostic accuracy, patient outcomes, and healthcare delivery, heralding a new era of innovation and personalized medicine in the field of neurology [18].

AI has become an invaluable tool in neurology diagnosis, offering unprecedented capabilities for the interpretation and analysis of complex neurological data [13]. AI algorithms trained on vast datasets of neuroimaging scans, such as MRI and CT scans, can detect subtle patterns and abnormalities indicative of neurological disorders with remarkable accuracy and efficiency [14]. In individuals with Parkinson's disease, MRI scans frequently display structural changes such as midbrain atrophy accompanied by third ventricle enlargement, tegmental atrophy, and irregularities in the superior contour of the midbrain [19]. Moreover, there is often an elevation in signal intensity within the midbrain and inferior olives, along with atrophy in the frontal and temporal lobes [19]. These alterations seen on MRI reflect the underlying neurodegenerative processes characteristic of Parkinson's disease and serve as valuable indicators for its diagnosis. Conversely, MRI images of healthy brains typically exhibit symmetrical structures, well-defined contrast, absence of atrophy, and normal ventricular size and shape [20]. These algorithms enable the early detection of conditions such as Alzheimer's disease, Parkinson's disease, stroke, and brain tumors, facilitating timely intervention and treatment planning [14,17]. Moreover, AI-driven diagnostic systems can integrate multiple data modalities, including clinical symptoms, genetic information, and biomarker profiles, to generate comprehensive diagnostic assessments tailored to individual patients [21]. By augmenting the expertise of healthcare professionals, AI empowers clinicians with enhanced diagnostic capabilities, enabling more accurate and timely diagnosis and ultimately improving patient outcomes and quality of care in neurology [21]. In this study, we aimed to develop an AI model capable of distinguishing between MRI scans of individuals with PD and those without PD. Utilizing cutting-edge machine learning algorithms and sophisticated image analysis methodologies, our study endeavors to furnish neurologists with a robust diagnostic instrument, capable of delivering precise and expedient assessments.

## Materials and methods

The study utilized MRI images obtained from a publicly available dataset sourced from a neuroimaging repository on Kaggle.com [22]. The dataset comprised 422 high-resolution MRI scans, with 221 scans representing patients diagnosed with PD and another 221 scans representing healthy controls.

To ensure the robustness and generalizability of the AI model, the dataset was randomly split into three distinct subsets: training, validation, and testing. Specifically, 80% of the dataset, totaling 337 images, was allocated to the training set. This training set facilitated the optimization of the AI model's parameters and the learning of underlying patterns associated with differentiating between PD and healthy controls. Subsequently, 10% of the dataset, comprising 42 images, was reserved for the validation set. The validation set served as an independent dataset for evaluating the model's performance during the training process and tuning hyperparameters to prevent overfitting. Iterative refinement of the model's architecture and optimization of training parameters were performed using the validation set to ensure optimal performance on unseen data. Finally, the remaining 10% of the dataset, consisting of 42 images, was designated as the testing set. The testing set remained untouched during the training and validation phases and was used to assess the model's performance on unseen data after training completion. Evaluation metrics such as accuracy, precision, recall (sensitivity), specificity, F1-score, and area under the curve (AUC) were computed based on the model's predictions on the testing set. The hyperparameters that were used were learning rate and number of layers. The learning rate controls the speed and stability of the learning process, while the number of layers determines the capacity and complexity of the neural network model. The learning rate acted as a hyperparameter that controls the other model parameters. For this model, learning rate={0.1 0.2,0.5,1.0} and num_layers={5,10,20,50,100}. 

The AI model employed in this study is a convolutional neural network (CNN), a specialized deep learning architecture renowned for its prowess in image recognition tasks. CNNs are particularly well-suited for analyzing complex visual data, such as MRI images, due to their hierarchical structure and ability to extract meaningful features at various levels of abstraction. By employing multiple layers of convolutional and pooling operations, CNNs can effectively capture intricate patterns and structures present in MRI images, enabling accurate classification of different neurological conditions.

The AI model was developed using state-of-the-art deep learning techniques, implemented using Python programming language and popular deep learning frameworks such as TensorFlow or PyTorch. Leveraging cloud computing resources, the model was trained efficiently within a duration of four hours and eight minutes. The use of cloud-based servers for model training ensured cost-free and environmentally friendly operation, aligning with sustainable practices. The methodology implemented in this study involved the acquisition and preprocessing of MRI images from a neuroimaging repository, partitioning the data into training, validation, and testing sets, development and training of the AI model using deep learning techniques, and evaluation of the model's performance using standard metrics on the testing set. Through this rigorous approach, we aimed to ensure the robustness, accuracy, and generalizability of the AI model for distinguishing between Parkinson's disease and healthy controls in MRI scans.

Ethical considerations

This study was deemed exempt from Institutional Review Board approval as it solely utilized a publicly available dataset, without involving direct interaction with human subjects. The dataset utilized in this study was obtained from openly accessible repositories, ensuring the highest level of personal data protection while upholding anonymity and confidentiality.

## Results

**Figure 1:**
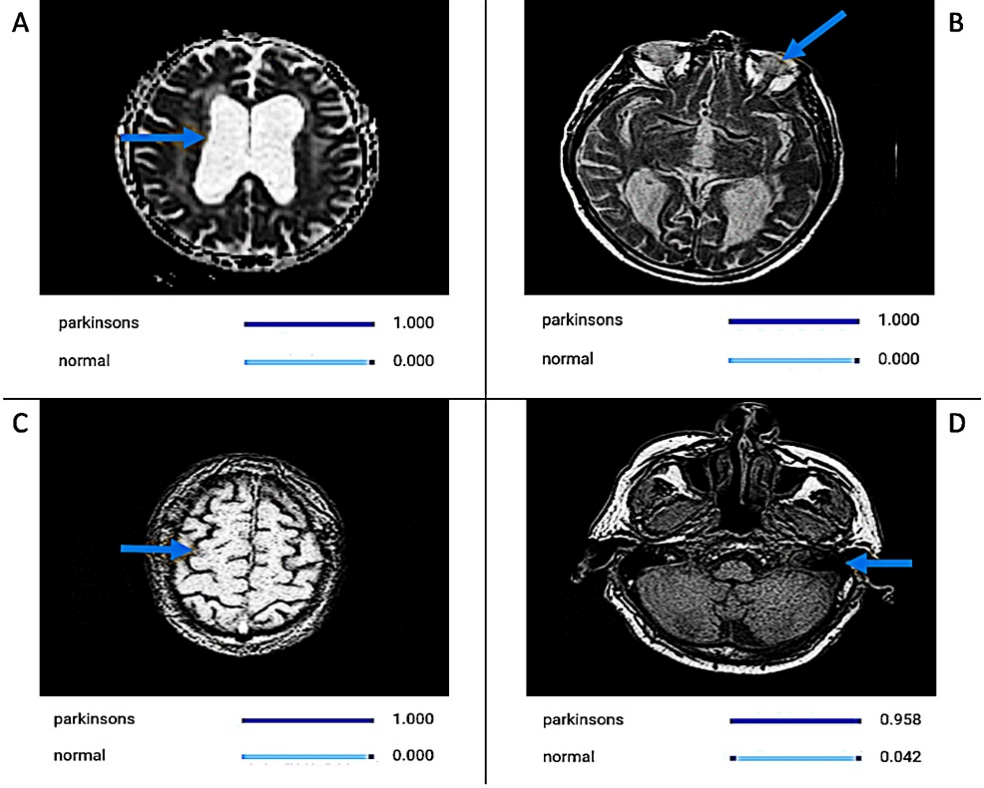
CNN Model Detecting MRI Images of Parkinson’s Disease The collection of MRI images from different patients highlights distinct characteristics of Parkinson’s Disease, including midbrain atrophy with third ventricle enlargement, tegmental atrophy, and an irregular superior contour of the midbrain. Moreover, signal elevation in the midbrain coupled with frontal and temporal lobe atrophy provides crucial diagnostic markers utilized by the AI model for precise recognition and diagnosis. Image A depicts signal elevation in the midbrain, image B depicts frontal lobe atrophy, image C depicts signal elevation in the midbrain, and image D depicts temporal lobe atrophy, and the blue arrows are used to point out these features. The values corresponding to the labels below the images represent the model calculating the likelihood of each possibility. For example, in image A where the model gives a value of 1.000 to parkinsons, it means that image A matched 100% of the model’s criteria for identifying an image of an MRI of a brain with Parkinson’s disease. CNN: Convolutional Neural Network; AI: Artificial Intelligence

**Figure 2:**
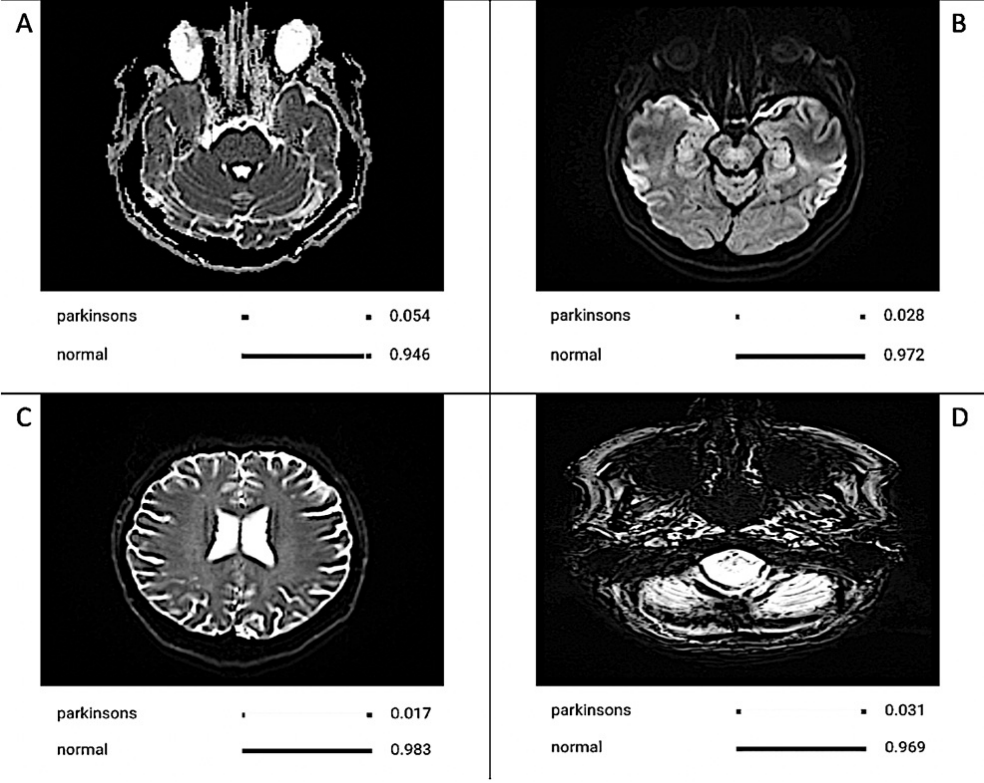
CNN Model Identifying MRI Scans Showing Normal Brains The array of MRI images from different patients underscores key features associated with a normal brain, including symmetrical structures, a distinct and well-defined contrast between gray and white matter, no signs of atrophy, and appropriately sized ventricles. These features offer essential diagnostic indicators employed by the AI model for accurate identification and diagnosis. Image A shows well-defined contrast between white and gray matter, image B shows symmetrical structure, image C shows no signs of atrophy, and image D shows appropriately sized ventricles. The values corresponding to the labels below the images represent the model calculating the likelihood of each possibility. For example, in image A where the model gives a value of 0.946 to normal, it means that image A matched 94.6% of the model’s criteria for identifying an image of an MRI of a normal brain. CNN: Convolutional Neural Network; AI: Artificial Intelligence

The AI model developed in this study exhibited exceptional performance in distinguishing between PD (Figure 1) and normal brains (Figure 2) based on MRI scans. Drawing upon a dataset comprising 221 MRI scans of Parkinson's disease (PD) patients and 221 MRI scans of healthy controls, our AI model showcased remarkable diagnostic accuracy and achieved outstanding performance across a range of evaluation criteria.

**Figure 3:**
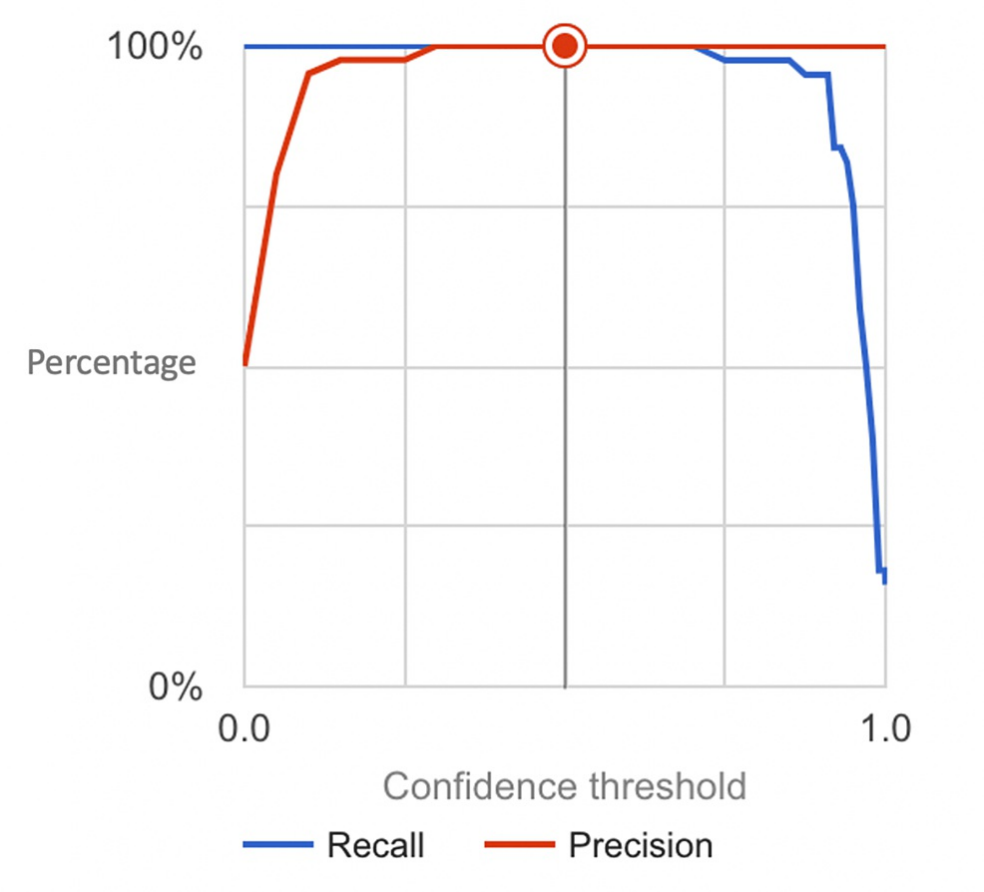
Precision-Recall Curve Depicting the Model's Discrimination between Parkinson’s Disease and Normal MRI Scans The graph showcases the precision and recall performance of the neural network model across various confidence thresholds.

**Figure 4:**
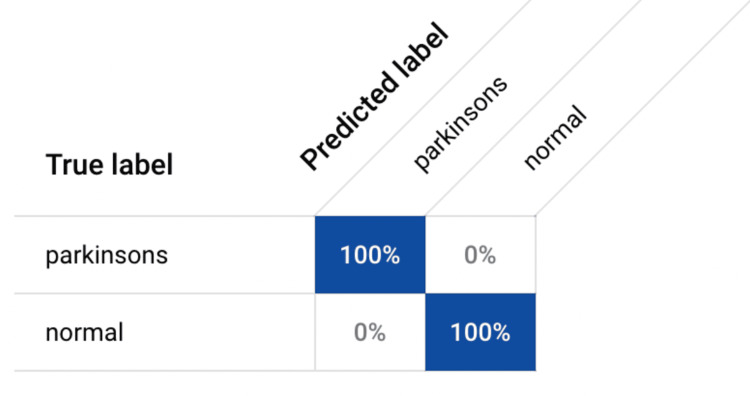
Confusion Matrix Different metrics, such as accuracy, precision, recall (sensitivity), specificity, and F-1 Score, were calculated based on information extracted from the confusion matrix.

**Figure 5:**
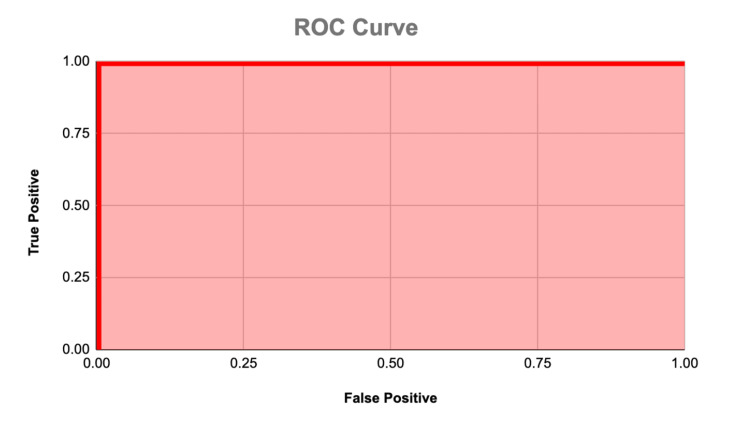
ROC Curve The thick red line represents the receiver operating characteristic (ROC) curve, which illustrates the trade-off between the true positive rate (sensitivity) and the false positive rate (1 - specificity) across different threshold values. The area under the ROC curve (AUC), which is the area shaded in red, quantifies the overall performance of the model, with higher values indicating better discrimination between the positive and negative classes, and our AUC was calculated to be 1.

Evaluation on the testing set revealed perfect scores across all performance metrics, including accuracy, precision, recall (sensitivity), specificity, and F1-score (calculated in Figure 3), all achieving a remarkable 100%. These metrics were obtained from the confusion matrix (Figure 4). These outstanding results underscore the model's capability to accurately differentiate PD cases from normal brain scans, highlighting its potential as a reliable diagnostic tool in clinical settings. The model's ability to achieve perfect scores across multiple metrics demonstrates its robustness and reliability, instilling confidence in its diagnostic accuracy and effectiveness in identifying PD cases with unparalleled precision. Furthermore, the analysis of receiver operating characteristic (ROC) curve (Figure 5) and the calculation of the AUC confirmed the model's exceptional discriminatory power. The AUC value was found to be 1, indicative of flawless classification performance. This suggests that the model can effectively distinguish between PD and normal brain scans, with optimal sensitivity and specificity. Visual inspection of the ROC curve further validated the model's outstanding performance across various classification thresholds, reinforcing its reliability in accurately diagnosing PD based on MRI scans.

## Discussion

In our investigation, we employed a cutting-edge CNN model to meticulously analyze MRI scans, discerning subtle nuances to differentiate between patients afflicted with PD and their healthy counterparts. The resulting performance of the model was nothing short of exceptional, demonstrating an unprecedented level of accuracy and precision that exceeded conventional benchmarks across a diverse array of stringent evaluation metrics, including accuracy, precision, recall, specificity, and F1-score. These remarkable outcomes unequivocally underscore the robustness and efficacy of the AI model in accurately diagnosing PD solely based on MRI scans, thus heralding its potential as an invaluable and indispensable diagnostic tool poised for seamless integration into real-world clinical settings. The attainment of such levels of accuracy and precision not only serves as a testament to the unwavering reliability and steadfast capability of the AI model but also signifies a pivotal breakthrough in the realm of PD diagnosis. Moreover, the model's exemplary performance extends beyond mere accuracy, exhibiting stellar proficiency across multiple evaluation criteria, including sensitivity and specificity, thereby fortifying its effectiveness in accurately distinguishing both positive and negative instances of PD with unmatched precision and fidelity. Such findings hold profound promise and significance, especially given the paramount importance of accurate and early diagnosis in PD management, as timely interventions are crucial for ameliorating symptoms, delaying disease progression, and ultimately enhancing patient outcomes and quality of life.

Furthermore, our rigorous examination of MRI scans also unveiled a multitude of notable and discernible characteristics intrinsically linked with PD, including midbrain atrophy, third ventricle enlargement, tegmental atrophy, and abnormalities in signal intensity [19]. These distinctive structural changes, corroborated by existing literature and established clinical paradigms, serve as poignant hallmarks that vividly elucidate the underlying neurodegenerative processes inherent to PD pathology [19]. The precise identification and delineation of these characteristic MRI features not only augment our diagnostic acumen but also offer invaluable insights into the intricate and complex neuroanatomical alterations intimately associated with PD [19]. Such revelations not only enrich our understanding of the pathophysiological mechanisms underpinning PD but also pave the way for the development of more targeted and efficacious therapeutic interventions aimed at mitigating disease burden and improving patient care and clinical outcomes.

Despite the promising results of our study, it is essential to acknowledge several limitations that may influence the interpretation and generalizability of our findings. Firstly, the performance of the AI model may be influenced by factors such as the size and diversity of the dataset, variations in image quality, and differences in patient demographics. While we employed rigorous preprocessing techniques and utilized a diverse dataset, including a sufficient number of PD and control subjects, the model's performance may still be subject to biases inherent in the data. Moreover, the AI model's performance in distinguishing PD from other neurodegenerative diseases or subtypes of PD was not explored in this study, limiting its applicability to broader clinical contexts. Additionally, the AI model's diagnostic accuracy may vary across different MRI scanners and acquisition protocols, as variations in imaging parameters can affect image quality and feature extraction. While we aimed to mitigate these variations by standardizing the preprocessing pipeline, variations in MRI protocols used across different clinical settings may still pose challenges to the model's generalizability. Furthermore, our study focused solely on MRI-based diagnosis of PD and did not incorporate other modalities such as PET or CSF biomarkers, which are commonly used in clinical practice for PD diagnosis and monitoring. Another limitation of our study is the lack of external validation in an independent dataset. While we employed cross-validation techniques to evaluate the model's performance robustness, validation in an external dataset would provide further evidence of the model's generalizability and reliability. Future studies should aim to validate the AI model's performance in diverse patient populations and clinical settings to ensure its applicability in real-world scenarios. One limitation of this study is the relatively small size of the dataset, comprising only 442 MRI images. While efforts were made to maximize the utility of available data through rigorous preprocessing and model optimization, the limited sample size may impact the generalizability of our findings. Additionally, a larger dataset would allow for greater variability in patient characteristics and imaging features, enhancing the robustness and reliability of the AI model. Further studies with larger datasets are warranted to validate the performance of the AI model in larger patient cohorts and clinical settings.

The findings of this study carry significant clinical implications for the diagnosis and management of PD. Through the seamless automation of MRI scan analyses, AI models not only augment but fundamentally redefine the diagnostic capabilities of clinicians, ushering in a new era of precision medicine. The exceptional performance of the AI model in accurately identifying PD cases based on MRI scans suggests its potential as a valuable diagnostic tool in clinical practice. By providing clinicians with a reliable and objective means of diagnosing PD at an early stage, the AI model can facilitate prompt interventions and treatment strategies, thereby potentially delaying disease progression and improving patient outcomes. Additionally, the identification of characteristic MRI features associated with PD pathology enhances clinicians' ability to recognize subtle neuroanatomical alterations indicative of the disease. Moreover, the integration of AI-driven diagnostic tools promises to mitigate diagnostic errors and significantly reduce inter-clinician variability, thereby elevating the overall quality and efficiency of patient care delivery to unprecedented heights [23]. This deeper understanding of PD's structural manifestations can inform treatment decisions and aid in monitoring disease progression over time. Furthermore, the integration of AI-driven diagnostic tools into clinical workflows has the potential to streamline diagnostic processes, reduce diagnostic errors, and optimize resource allocation in healthcare settings [24]. Thus, the widespread adoption of AI technology within neuroimaging offers a tantalizing glimpse into a future wherein neurological disorders are diagnosed, monitored, and managed with unparalleled precision, efficacy, and compassion. Ultimately, the clinical implications of this study underscore the transformative role of AI technology in improving the diagnosis and management of PD, thereby enhancing patient care and quality of life.

Our study constitutes a significant advancement in the field of neurology, representing a substantial progression from prior research endeavors that have integrated AI into neurological diagnostics. Unlike previous studies that have primarily explored AI's utility in diagnosing various neurological conditions such as Alzheimer's disease, multiple sclerosis, and brain tumors, our investigation hones in on PD, a prevalent and complex neurodegenerative disorder [25,26]. By focusing our efforts on PD diagnosis, we aim to address a critical gap in the existing literature and provide a targeted solution to a pressing clinical need. In comparison to previous research, our study's focus on PD diagnosis and the development of AI-driven diagnostic tools represents a significant step forward in clinical neurology [27]. Through the meticulous deployment of advanced machine learning algorithms and sophisticated image analysis techniques, our study endeavors to develop a diagnostic tool that offers unparalleled precision and efficiency in detecting PD from MRI scans. This study not only adds to the growing body of knowledge on AI applications in neurology but also underscores the importance of tailored solutions for specific neurological disorders [25]. By delving into the intricate nuances of PD diagnosis, we seek to pave the way for more nuanced and effective clinical interventions, ultimately improving patient outcomes and enhancing the quality of neurological care. These insights not only contribute to the elucidation of disease mechanisms but also hold the potential to inform the development of novel therapeutic strategies targeting PD [28]. In essence, our study represents a pivotal step forward in leveraging AI technologies to address the multifaceted challenges posed by neurological disorders. By combining cutting-edge AI methodologies with a targeted focus on PD diagnosis, we aim to catalyze advancements in neurology and pave the way for more personalized and effective approaches to patient care. Through our research endeavors, we aspire to empower clinicians with the tools and insights needed to navigate the complexities of PD diagnosis and treatment, ultimately ushering in a new era of precision medicine in neurology.

Moreover, our study goes beyond mere diagnostic accuracy by shedding light on the characteristic MRI features associated with PD pathology. Through the elucidation of these neuroanatomical alterations, we enhance our understanding of the underlying disease processes, paving the way for further research and the development of more targeted therapeutic interventions [29]. By elucidating the intricate interplay between structural changes in the brain and PD pathology, our findings contribute to a deeper comprehension of the disease's pathophysiology [2]. This comprehensive approach not only advances our scientific understanding but also holds promise for translating research findings into clinical practice, ultimately improving patient care and clinical outcomes in the realm of neurology. In essence, our study represents a significant leap forward in leveraging AI technologies to address the complex challenges posed by neurological disorders, marking a pivotal moment in the quest for improved patient care and clinical management strategies.

## Conclusions

In summary, this study highlights the significant potential of AI technology in the realm of neurology, particularly regarding the diagnosis and characterization of PD through MRI scans. Through the utilization of a CNN model trained on an extensive dataset of MRI images, our findings underscore the remarkable performance of AI-driven diagnostic tools in accurately discerning PD cases from healthy controls. The exceptional accuracy, precision, and efficacy demonstrated by the AI model emphasize its role as a valuable asset in clinical settings, providing clinicians with a robust tool for the early and precise diagnosis of PD. Additionally, our investigation has shed light on the distinct MRI features associated with PD pathology, offering valuable insights into the underlying neuroanatomical alterations driving the disease process. These discoveries not only deepen our understanding of PD but also lay the groundwork for the development of more targeted and effective therapeutic interventions. Looking ahead, continued research and innovation in AI-driven neuroimaging hold the promise of further enhancing diagnostic capabilities, advancing patient care, and ultimately alleviating the burden of neurodegenerative disorders like PD. By harnessing the capabilities of AI technology, we have the potential to revolutionize the field of neurology and significantly improve the quality of life for individuals affected by neurological conditions.
